# Adoption of Telemedicine for Type 1 Diabetes Care During the COVID-19 Pandemic

**DOI:** 10.1089/dia.2021.0080

**Published:** 2021-09-01

**Authors:** Joyce M. Lee, Emily Carlson, Anastasia Albanese-O'Neill, Carla Demeterco-Berggren, Sarah D. Corathers, Francesco Vendrame, Ruth S. Weinstock, Priya Prahalad, Guy Todd Alonso, Manmohan Kamboj, Daniel J. DeSalvo, Faisal S. Malik, Roberto Izquierdo, Osagie Ebekozien

**Affiliations:** ^1^ C.S. Mott Children's Hospital, University of Michigan, Ann Arbor, Michigan, USA.; ^2^ T1D Exchange, Boston, Massachusetts, USA.; ^3^ University of Florida, Gainesville, Florida, USA.; ^4^ Rady Children's Hospital/University of California, San Diego, California, USA.; ^5^ Cincinnati Children's Hospital Medical Center, University of Cincinnati College of Medicine, Cincinnati, Ohio, USA.; ^6^ University of Miami Miller School of Medicine, Miami, Florida, USA.; ^7^ SUNY Upstate Medical University, Syracuse, New York, USA.; ^8^ Lucile Packard Children's Hospital/Stanford University, Palo Alto, California, USA.; ^9^ Barbara Davis Center, University of Colorado, Aurora, Colorado, USA.; ^10^ Nationwide Children's Hospital, Columbus, Ohio, USA.; ^11^ Texas Children's Hospital, Baylor College of Medicine, Houston, Texas, USA.; ^12^ University of Washington, Seattle, Washington, USA.; ^13^ Bower School of Population Health, University of Mississippi Medical Center, Jackson, Mississippi, USA.

**Keywords:** COVID-19, Type 1 diabetes, Diabetes, Telemedicine, Virtual, Telehealth

## Abstract

***Background:*** We describe the utilization of telemedicine visits (video or telephone) across the type 1 diabetes (T1D) Exchange Quality Improvement Collaborative (T1DX-QI) during the COVID-19 pandemic. Metrics, site-level survey results, and examples of interventions conducted to support telemedicine in T1D are shown.

***Materials and Methods:*** Thirteen clinics (11 pediatric, 2 adult) provided monthly telemedicine metrics between December 2019 and August 2020 and 21 clinics completed a survey about their telemedicine practices.

***Results:*** The proportion of telemedicine visits in T1DX-QI before the pandemic was <1%, rising to an average of 95.2% in April 2020 (range 52.3%–99.5%). Three sites initially used mostly telephone visits before converting to video visits. By August 2020, the proportion of telemedicine visits decreased to an average of 45% across T1DX-QI (range 10%–86.6%). The majority of clinics (62%) performed both video and telephone visits; Zoom was the most popular video platform used. Over 95% of clinics reported using CareLink™, Clarity^®^, Glooko™, and/or t:connect^®^ to view device data, with only one center reporting automated data upload into the electronic medical record. The majority of centers had multidisciplinary teams participating in the video visits. All sites reported reimbursement for video visits, and 95% of sites reported coverage for telephone visits early on in the pandemic.

***Conclusions:*** There was rapid adoption of telemedicine in T1DX-QI during the COVID-19 pandemic. Future insurance reimbursement for telemedicine visits and the ideal ratio of telemedicine to in-person visits in T1D care remain to be determined.

## Introduction

A number of studies have provided evidence for the utility of telemedicine in diabetes care,^1–7^ including improved provider and patient satisfaction and improved engagement in visits. However, critical barriers have historically impeded its adoption and dissemination as a sustainable model for delivering diabetes care. These include provider attitudes toward telemedicine, patient and family lack of access to technology, device costs, suboptimal health care technology infrastructure to support telemedicine, and strict reimbursement criteria by third party payers.^8^

The initial lockdown orders that abruptly limited in-person health services delivery across the U.S. health system at the onset of the COVID-19 pandemic in March 2020 created the opportunity for providers, health systems, and third party payers to transition en masse to the use of telemedicine for their patients with diabetes. Published case studies recently described the novel use and adoption of telemedicine during the COVID-19 pandemic for managing patients with new onset type 1 diabetes (T1D) in the United States^9^ and internationally.^10^

T1D Exchange Quality Improvement Collaborative (T1DX-QI) (https://t1dexchange.org/quality-improvement/collaborative/) is a network of 31 pediatric and adult diabetes centers from many different regions across the United States committed to sharing best practices with the aim of improving outcomes for people with diabetes. We wished to describe and understand the shift to telemedicine as a U.S. health care enterprise during the course of the pandemic. Therefore, our objective was to describe the adoption and utilization of telemedicine for T1D care across leading U.S. health systems participating in the T1DX-QI. We describe the metrics of telemedicine adoption, the results of a telemedicine survey, and share best practices performed by centers to effectively implement, support, and maintain this new model of care.

## Materials and Methods

**FIG. 1.**
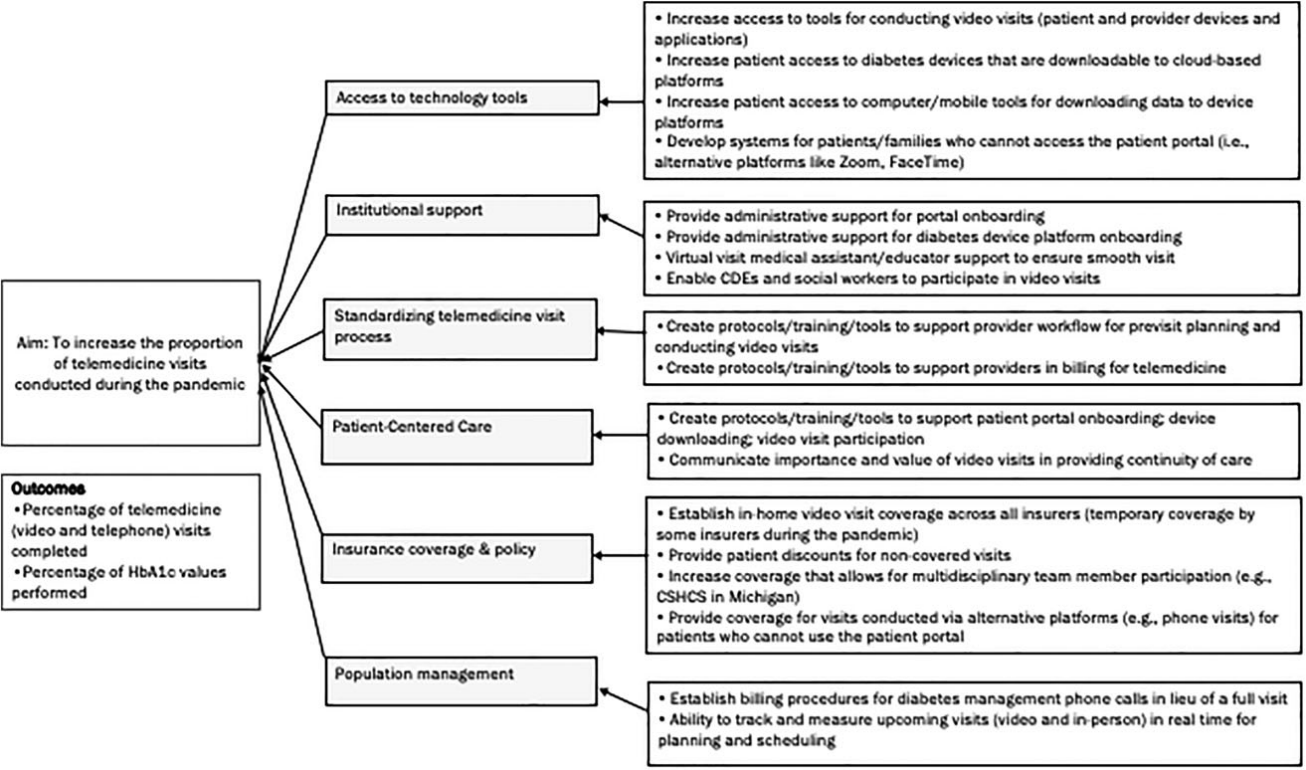
Telemedicine improvement key driver diagram. CDE, certified diabetes educator.

At the start of the pandemic, when the need for telemedicine became imperative, the T1DX-QI focused on telemedicine adoption as a quality improvement initiative. A key driver diagram (KDD), a graphic model for describing components required to accomplish an aim (Fig. 1), was developed with input from participating clinics. Centers began meeting monthly through virtual meetings/collaborative calls to share progress and best practices. Resources, including protocols and tools, were shared asynchronously with T1DX-QI in an online common.

A subset of sites provided the monthly total number of T1D visits (in clinic, telephone visit, or video visit) between December 2019 and August 2020. They also provided the monthly number of A1cs performed in their patient population to capture possible missed laboratory work (especially HbA1c) as a result of the pandemic.

Twenty-one clinics (16 pediatric and 5 adult) answered a center-level survey that covered topics related to the key drivers on the KDD, including access to technology tools, institutional support, standardizing telemedicine visit processes, patient-centered care, insurance coverage/reimbursement policies, and population management (Table 1). Telemedicine visits were defined as video visits or telephone visits. Video visits were defined as face-to-face medical visits that occurred using videoconferencing software. Telephone visits were defined as a billable telephone call conducted in the place of a planned medical visit that could not take place face to face because of the COVID-19 pandemic. Phone calls that occurred between scheduled visits for insulin dose adjustments or medical advice were not included in the definition. Table 2 gives examples of the interventions developed to support telemedicine best practices. This study was approved by Western IRB and each clinic obtained its own approvals per institutional policy.

**Table 1.** Results from T1DX-QI Telemedicine Survey

	No. of centers	% of centers
Tools for conducting telemedicine visits		
Videoconferencing software only	7	33%
Phone calls only	1	5%
Both videoconferencing software and phone calls	13	62%
Types of software used		
Videoconference	9	43%
Zoom	13	62%
FaceTime^®^	4	19%
Microsoft teams	2	10%
Doximity	3	14%
WebEx^®^	4	19%
BlueJeans	1	5%
Health system developed technology	1	5%
Google Duo	1	5%
WhatsApp™	1	5%
Insurance coverage^a^		
Telephone visit coverage	19	95%
Video visit coverage	20	100%
Tools used for accessing diabetes device data		
Medtronic (CareLink™)	21	100%
Tandem t:slim (t:connect^®^)	21	100%
Dexcom (Clarity^®^)	21	100%
Glooko™	20	95%
Tidepool™	8	38%
Abbott Libre (LibreView™)	5	24%
Nightscout	1	5%
Workflow for EHR download		
Either clinic staff or provider captures download	7	33%
Clinic staff captures download	11	52%
Provider captures download	2	10%
Data integrated into the EHR and clinic staff provide support	1	5%
Non-MD providers participating in telemedicine^a^		
RD	19	95%
CDCES	18	90%
Social worker	16	80%
RN	12	60%
Psychologist	3	15%
MD and diabetes team workflow^a^		
Separately by phone and in conjunction with video visit	5	25%
Separately for both	11	55%
In conjunction for video visits	4	20%
Workflow available to support care components		
Participation of interpreters	19	90%
Obtaining patient laboratories	13	62%
Depression screening	8	38%
Virtual device training^a^		
Provides insulin pump training using telephone or video	20	100%
Provides CGM training using telephone or video	14	70%
Institutional goal for the overall percentage of diabetes telemedicine visits		
Unsure	12	57%
0%–20%	2	10%
21%–40%	4	19%
41%–50%	3	14%

^a^ 20 clinics reported.

CDCES, certified diabetes care and education specialist; CGM, continuous glucose monitoring; EHR; RD, registered dietitian; RN, registered nurse; T1DX-QI, T1D Exchange Quality Improvement Collaborative.

**Table 2.** List of Site Interventions According to Driver

Driver	Site examples
Access to technology tools	Instructions for uploading device data sent to patients through email or My Chart in advance of visits (four clinics)
	CDCES, MA, RD provide previsit support to patients regarding device uploading (five clinics)
	Virtual pump onboarding classes executed (three clinics)
	Cameras added to office and clinic computers to facilitate telemedicine appointments (four clinics)
Institutional support	Quick institutional adoption of telemedicine video visits using a variety of platforms (Zoom, WebEx, WhatsApp, Facetime) (four clinics)
	Variety of processes developed and communicated to staff by institutions, including:
	Providers conducting telemedicine visits from their homes (one clinic)
	New role—telehealth business manager—created to support telemedicine adoption (one clinic)
	Integrating Zoom directly into Epic (two clinics)
Standardizing telemedicine visit process	Coding SOP developed to ensure standardized coding (three clinics)
	Redistribution of staff roles and responsibilities to assist with telemedicine visits (five clinics)
	Weekly huddle with dedicated time to discuss clinic flow and opportunities for improvement (one clinic)
Patient-centered care	Depression screening conducted through virtual forms or verbally with RN (two clinics)
	Workflow allowed for DE, RD, psychologist, or social worker to join telemedicine visit if necessary (four clinics)
	Interpreter services available during telemedicine visit (three clinics)
	Obtained grant funding to provide Libre sensors (>1000 U) to patients without CGM access. Linking device to LibreView account facilitated data sharing before telehealth visits (one clinic)
Insurance coverage and policy	Institutional decision to see all patients through telemedicine irrespective of ability to pay. Swift efforts made to have providers approved for insurance coverage from adjoining states so all patients could be seen. (one clinic, Ohio)
	Executed contract with local school district that established partnership to provide direct school telehealth (one clinic, Florida)
	COVID-19 enabled telephone coverage addition to existing telemedicine parity law allowing reimbursement for video appointments (one clinic, California)
Population management	Expanded focus on LTFU tracking, including:
	Reports generated weekly to track (two clinics)
	Definition expanded to include: no shows, cancellations, and those with no future visit scheduled due to workflow challenges (three clinics)
	Staff redeployed to follow-up with LTFU patients (three clinics)

DE, diabetes educator; LTFU, lost to follow-up.

## Results

A total of 13 (11 pediatric and 2 adult) clinics provided visit level data about telemedicine metrics and a total of 21 clinics (16 pediatric and 5 adult) completed the survey. The Appendix provides a list of the participating centers.

**FIG. 2.**
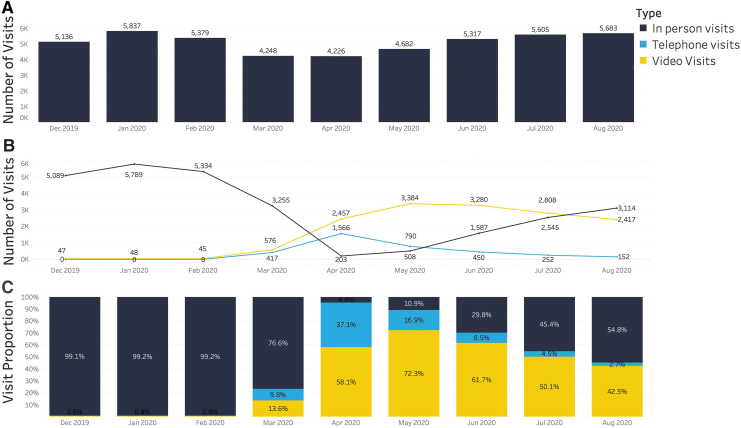
Monthly visit volumes for T1DX-QI from December 2019 to August 2020. **(A)** Total number of overall visits. **(B)** Total number of clinic, telephone, and video visits. **(C)** Proportion of clinic, telephone, and video visits. T1DX-QI, T1D, Exchange Quality Improvement Collaborative.

Figure 2A shows the total number of overall visits by month for T1DX-QI. Compared with the prepandemic average number of visits (*n* = 5450), there was a 22% reduction in overall visits during the months of March and April 2020, followed by an increase in overall visit volumes to prepandemic levels with a mixture of telemedicine and in-person visits by June 2020. Figure 2B shows the total number of clinic, telephone, and video visits by month, and Figure 2C shows the proportion of clinic, telephone, and video visits by month. Overall, during the prepandemic months of December 2019 to February 2020, fewer than 1% of visits were conducted through telemedicine, but in April 2020, telemedicine accounted for 95.2% of visits across T1DX-QI.

**FIG. 3.**
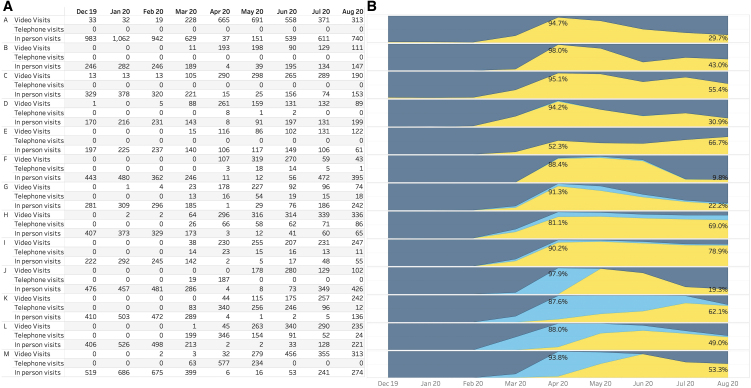
Monthly visit volumes for 12 individual sites from T1DX-QI from December 2019 to August 2020 **(A)**. Total number and proportion of clinic, telephone, and video visits by site **(B)**.

Figure 3A shows the monthly volume, and Figure 3B shows the proportion of clinic, telephone, and virtual visits by site during the pandemic. Among the six sites that had performed video visits before the pandemic, the number of visits per month ranged from 1 to 33 visits; seven sites had not performed any video visits before the pandemic, and none of the sites had performed any billable telephone visits. All centers reached their peak proportion of telemedicine visits in April 2020. By April 2020, the percentage of visits conducted as telemedicine visits across centers ranged from 52.3% to 99.5%. There were at least three centers that achieved this through a ramp-up of majority telephone visits first during the month of April 2020. One site gradually increased their proportion of video versus telephone visits over a 4-month period, whereas the two other sites converted to telemedicine more quickly. From April 2020 onward, the proportion of telemedicine visits dropped over time, and by August of 2020, the proportion of visits conducted by telemedicine was 45% across T1DX-QI (range 10%–86.6%).

**FIG. 4.**
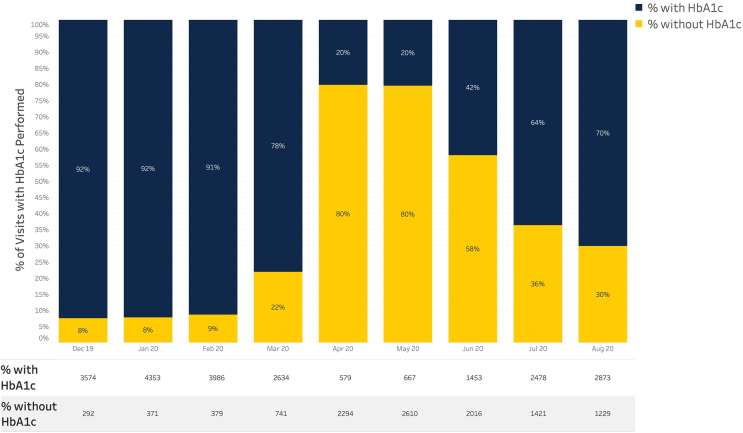
Monthly number of patients with and without HbA1c values.

Figure 4 shows a subset of nine clinics provided information about deferred HbA1c laboratories during the pandemic. Over 60% of visits had missing laboratories in April 2020, but this percentage decreased with time as clinics developed workflows for obtaining laboratory results.

Table 1 gives the results of the survey across 21 sites. The majority of clinics (62%) performed both video visits and phone calls, with just one center only performing phone calls. Zoom was the most popular platform used (62%), followed by a variety of other tools.

Over 95% of clinics reported using the four major data integration platforms (CareLink™, t:connect^®^, Clarity^®^, and Glooko™) to view diabetes data. There was just one center that had diabetes data from platforms integrated into the electronic medical record system. The rest of the centers required provider or clinic staff to capture the download, and at least 9 of the 21 centers had physicians or advanced practice providers involved in the process.

The majority of centers had diabetes educators, registered dietitians, and social workers involved in the telemedicine visits, but fewer centers had psychologists participating. To provide multidisciplinary diabetes care, most diabetes team members met with patients separately from the medical provider visit.

Nearly all clinics had workflows to incorporate interpreters, over half had workflows to obtain patient laboratory results, but just a few had had a system for conducting depression screening. The majority of centers were able to adapt and provide continuous glucose monitors (CGMs) (100%) and insulin pump training (70%) during the pandemic.

Regarding insurance coverage of telemedicine visits, 100% of sites reported that there was reimbursement of video visits, and 95% of sites reported that there was reimbursement of telephone visits early on during the pandemic.

When asked what their institutional goal was for telemedicine visits overall, a large number of centers (*n* = 12) could not yet articulate a long-term goal for telemedicine visits. However, a subset of centers did articulate a desired goal ranging in descending order of 21%–40%, 41%–50%, and 0%–20%. Table 2 summarizes the interventions, organized by key drivers, conducted by sites enabling the adoption of telemedicine in their centers.

## Discussion

We have described the rapid adoption of telemedicine visits for clinics providing medical care to T1D patients across the United States during the start of the COVID-19 pandemic. The precipitous drop in care delivery during the month of March because of the initial lockdown that occurred led to a significant amount of delayed care and represented a major financial threat to clinics. Faced with these constraints, the T1DX-QI clinics rapidly accelerated adoption of telemedicine visits from ∼1% before the pandemic to 94.7% of all visits within a month's time, completing a near total digital transformation of T1D health care delivery early in the pandemic.

A number of drivers guided this rapid adoption, but two of the most critical drivers were expanded insurance coverage for telemedicine visits and the ability to utilize a broad range technology tools to support the visits.^11^ In early March 2020, the Centers for Medicare & Medicaid Services (CMS) issued the 1135 waiver for the period of the COVID-19 public health emergency,^12^ supported broader coverage of providers, patients, and services, allowed for treatment of all diagnoses, allowed patients to access services from their homes, did not require a pre-existing relationship with the patient, waived the requirement for a video component, and provided reimbursement of visits at the same rates as in-person clinic visits.^11^ Furthermore, the policy allowed for the use of any “nonpublic facing communication product” available to communicate with patients during the pandemic, allowed providers to practice across state lines, and waived potential penalties for HIPAA violations against health care providers using video platforms.^13^

Because of these changes, private insurers adopted similar guidelines, allowing clinics to provide medical care remotely.^11^ This enabled health systems and providers to quickly onboard their patient populations to telemedicine en masse and offer diabetes care that otherwise would not have occurred because of the pandemic. Based on the site-specific uptake across centers, the ability to bill for telephone visits was critically important for three of the clinics that relied almost exclusively on telephone visits during the first full month of the pandemic, until they could adopt video tools. Telephone visits provided opportunities to reach patients who might otherwise have had difficulty with accessing virtual visits, for example, do not have a computer, tablet, or smartphone, or do not have internet access.

Across T1DX-QI, the COVID-19 pandemic forced a rapid nearly comprehensive conversion of outpatient diabetes care to telemedicine, and it was largely successful for these U.S. diabetes clinics based on the continuity of care provided. Their immediate capacity to support this new model of care demonstrates flexibility and facilitation of the widespread use of diabetes devices and diabetes data-sharing platforms that were in place before the pandemic. As stay-at-home orders were relaxed across the country, clinics resumed in-person care but maintained sizable fractions of care through this new vehicle. However, it was interesting to discover a range of telemedicine use, even between clinics in the same state that would have been influenced by similar state and private insurer policies. For example, centers in one state reported telemedicine rates of 9.9% at one site and 22.2% at another; in another state, centers reported telemedicine rates of 19.3% at one site and 62.1% at another.

Although many aspects of diabetes care can be performed virtually, in-person clinic visits are important for evaluating vital signs, growth, weight, and performing a physical examination to support an evaluation of diabetes-related complications and comorbidities. For many patients, it may only be necessary to do these once or twice yearly, whereas some of the cognitive aspects of diabetes care need to take place more frequently and, therefore, lend themselves to being performed by telephone or videoconference. By late summer, many patients had already been seen at least once by telemedicine, and patients and providers alike may have been aiming to see patients in person at least once before the anticipated winter COVID-19 resurgence. Also important, insurance reimbursement may have been a barrier, as several large insurers did not continue to provide coverage for telemedicine visits despite the continuation of CMS public health provisions. The likelihood of health systems or providers to maintain certain levels of telemedicine visits may also be influenced by the work of telemedicine visits. Many aspects of telemedicine are convenient for patients and providers including reduced travel and time to complete a visit but the pre-visit preparation, which includes diabetes device data downloading and obtaining laboratories, adds new steps that are more challenging to support remotely. In addition, the prevalence of COVID-19 varied between communities with a spectrum of restrictions between campuses and jurisdictions. Finally, and most importantly, patients may have personal preferences about the patient experience of a telemedicine visit and may have differing opinions about the perceived value of the telemedicine visit compared with a clinic visit. These factors are reflected variably across sites regarding what they consider to be the ideal proportion of telemedicine visits. Further research is needed to understand these factors and preferences influencing the uptake and sustainability of this model of care.

A hallmark of T1D care is involvement of a multidisciplinary team and, fortunately, the majority of centers were able to continue this multidisciplinary model of care, using new virtual workflows. Certified diabetes care and education specialists, dietitians, nurses, social workers, and psychologists were successfully incorporated into telemedicine visits, although sometimes asynchronously from the medical provider visit. Centers were largely able to continue diabetes education virtually. This may have been due to the broadened coverage of virtual behavioral health and patient education services^14^ because of the COVID-19 emergency public health provisions.^15^ However, the ability of teams to maintain telemedicine as a viable model for delivery care may change if telemedicine payment models do not persist beyond the pandemic.

Downloading and reviewing diabetes data are essential for making effective medical management decisions in diabetes. With the new virtual model, patients and families had to perform their downloads at home rather than having clinic personnel do it for them in the clinic. Centers adapted to this new system, but the fact that just one site had direct integration of diabetes data into the electronic health records (EHR) workflow and others involved providers in the data downloading process suggests that much work needs to be done to reduce the difficulties in viewing and downloading data from multiple devices. Clinic staff had to be available to help patients troubleshoot at home downloading. The complexity of needing to use multiple platforms and lack of interoperability between devices, platforms, and the electronic medical record EHR^16^ are barriers that are unlikely to be solved in the near future.

We are unaware of studies to date that have documented the uptake of telemedicine during the pandemic for the total population of patients with T1D seen at multiple centers across the United States, and for a time frame that extends well beyond the start of the pandemic. A recent study of diabetes centers described adaptations of care delivery in international diabetes clinics in response to COVID-19, but consisted of just nine centers and did not provide quantitative information about the uptake of telemedicine or site-specific practices.^10^ Another publication described quality improvement activities used to employ telemedicine services at a National Health Service Hospital in the United Kingdom during the pandemic.^17^ However, this reports the data and experience of just one hospital in a government-supported health system where most visits were conducted by phone rather than video visits, the observation period was relatively brief (6 weeks), early in the pandemic, and the study did not focus on a diabetes population.

T1D is a medical condition most suited for the telemedicine format, given the growing number of patients who use meters, smart pens, CGM systems and insulin pumps, the ability to upload the data generated by these devices from home, and the ability for diabetes data to guide medical decision making and adjustments to insulin dosing. Based on anecdotal reports from centers, there is enthusiasm about continuing to perform telemedicine but future post-pandemic coverage of telemedicine visits is uncertain. Even if insurers continue reimbursement for telemedicine visits, payment parity, which is defined as telemedicine visits being reimbursed at the same rate as in-person clinic visits, may not be mandated, which is a financial disincentive to health systems, and could jeopardize the continued use of this effective, convenient, and patient-centered care tool.

Although the rapid adoption of telemedicine highlights the impressive ability of T1DX-QI clinics to quickly adapt to the challenges of the COVID-19 pandemic, strategies on how to best implement this novel modality in the most equitable manner remain a concern. Making health equity integral to the implementation of telemedicine programs is key to ensuring that all can benefit from its use going forward.^18^ Lack of access to the technology and unequal coverage of video visits and, in particular, telephone visits across populations may further exacerbate health disparities in diabetes care.

## Conclusions

Physicians and insurers have adopted telemedicine with remarkable speed, and this new tool has been rapidly employed in diabetes clinics in the T1DX-QI. Future studies in this network will assess the effectiveness and outcomes of telemedicine visits during this pandemic in different patient populations.

## Appendix

**Appendix A:** List of Clinics Participating Clinics in T1D Exchange Telemedicine Study 2021

Clinic Name	PI Names
1. Barbara Davis Center for Childhood Diabetes, Children's Hospital	G. Todd Alonso
2. Barbara Davis Center for Diabetes, Adult	Sarit Polsky
3. Baylor College of Medicine, Texas Children's Hospital	Daniel DeSalvo
4. Children's Hospital of Los Angeles	Brian Miyazaki
5. Children's Mercy Hospital	Mark Clements
6. Cincinnati Children's Hospital Medical Center	Sarah Corathers
7. Cook Children's Medical Center	Susan Hsieh
8. Nationwide Children's Hospital	Manmohan Kamboj
9. NYU Langone Adult	Lauren Golden
10. NYU Langone Health Pediatric	Mary Pat Gallagher
11. Penn Rodebaugh Diabetes Center, Penn Medicine Adult	Ilona Lorincz
12. Rady Children's Hospital	Carla Demeterco-Berggren
13. Seattle Children's Hospital	Faisal Malik & Alissa Roberts
14. Stanford Adult Diabetes	Marina Basina
15. Stanford Pediatrics, Lucille Packard Children's Hospital	Priya Prahalad
16. SUNY Upstate Medical University, Joslin Diabetes Adult	Ruth Weinstock
17. SUNY Upstate Medical University, Joslin Diabetes Pediatric	Roberto Izquierdo
18. Univ. of Florida Diabetes Institute Pediatric	Laura Jacobson
19. Univ. of Miami, Miller School of Medicine Adult	Francesco Vendrame
20. Univ. of Miami, Miller School of Medicine Pediatric	Janine Sanchez
21. Univ. of Mich. Hospitals-Mich. Med, C.S. Mott Children's Hospital	Joyce Lee
